# Diet, nutrition, and climate: historical and contemporary connections

**DOI:** 10.3389/fnut.2024.1516968

**Published:** 2024-11-27

**Authors:** Kathrin M. Demmler, M. Ann Tutwiler

**Affiliations:** ^1^Knowledge Leadership, Global Alliance for Improved Nutrition, Berlin, Germany; ^2^Board Chair, Global Alliance for Improved Nutrition, Washington, DC, United States

**Keywords:** nutrition, climate change, food systems, diets, sustainability, policy

## Abstract

This paper reviews the past global nutrition efforts, particularly those led by the Global Alliance for Improved Nutrition (GAIN), at the critical intersection of nutrition and climate change. Despite progress in tackling malnutrition and promoting sustainable food systems, significant challenges remain, especially in regions like Sub-Saharan Africa and South Asia, where micronutrient deficiencies persist. The paper underscores the urgent need to integrate nutrition into climate strategies and strengthen food system resilience. Initiatives like the Scaling Up Nutrition (SUN) movement, the Food Systems Dashboard, and GAIN’s public and private sector partnerships at the local levels have contributed to transforming food systems. However, there is an urgent need for more robust policies that effectively align nutrition, climate, and equity goals. Looking ahead, we advocate for increased financial investment, improved policy frameworks, and innovations in technology and data monitoring to drive sustainable food system transformations. We further underscore the importance of addressing micronutrient deficiencies, promoting biodiversity, and developing healthier crops to support climate-smart agriculture. Achieving resilient, equitable, and sustainable food systems over the next years will depend on collaborative efforts across sectors and stakeholders.

## 1 Introduction

The narrative around nutrition has evolved significantly, shifting from a focus on infection and mortality to acknowledging its critical role in overall health and development. Early efforts targeted undernutrition, but the approach has since broadened to include overweight and obesity and related non-communicable diseases, as well as multisectoral interventions and food systems, recognizing their impact on both human health and the environment.

Today, the link between nutrition and climate change is central due to the profound impacts that food production and consumption have on the environment and vice versa. Food systems contribute significantly to environmental degradation through practices such as deforestation, greenhouse gas emissions, and excessive water use in agriculture. These processes drive biodiversity loss and exacerbate global warming, with food production, processing, and transportation being key contributors to these environmental pressures (1–3). However, food processing also plays an essential role in reducing food waste, which is critical for minimizing environmental impact across multiple indicators (4). At the same time, climate change poses substantial risks to food production. Increasingly frequent extreme weather events, shifting agricultural zones, and changing rainfall patterns threaten food security and nutrition, with direct impacts on food supply, quality and safety that can lead not only to food insecurity but also to dietary shifts toward more processed foods (5–8). This bidirectional relationship between food systems and climate change underscores the urgency of addressing climate change through sustainable food and nutrition practices.

A growing body of research and evidence, including reports by the United Nations and the Intergovernmental Panel on Climate Change, has emphasized the need for integrated approaches to mitigate climate change while improving nutrition. However, integrating nutritional goals with climate objectives remains a complex challenge. Particularly in regions like Sub-Saharan Africa and South Asia, where undernutrition and high rates of micronutrient deficiencies persist, rising influence of Western dietary patterns, including increased consumption of red meat, is driving obesity and non-communicable diseases. These shifts complicate efforts to promote sustainable and nutritious consumption patterns (9–12).

This perspective paper reflects on past efforts at the intersection of nutrition and climate, using the lens of work undertaken by the Global Alliance for Improved Nutrition (GAIN) during this period. It also explores future approaches needed to address ongoing challenges and highlights potentially impactful strategies moving forward.

## 2 Historic overview and current state

### 2.1 Review of past trends and key milestones

The 2008 food price crises, where rice prices rose by 300% (13) and wheat and maize doubled (14) exposed the vulnerability of food security to price volatility. This highlighted the need for resilient food systems to ensure global food security amidst fluctuating markets.

#### 2.1.1 Nutrition and growth

Launched at the 2010 UN Millennium Development Goals Summit, the Scaling Up Nutrition (SUN) movement emphasizes the critical 1,000-day period from conception to a child’s second birthday. Driven by the 2008 Lancet series on Maternal and Child Undernutrition (15), it promotes nutrition-specific and nutrition-sensitive interventions through country-led, multi-actor efforts involving governments, donors, civil society, businesses, and academia. This marked a shift from focusing solely on hunger and human rights to highlighting the economic impacts of malnutrition, including micronutrient deficiencies, wasting, stunting, obesity and the rising burden of non-communicable diseases (16, 17).

Since its inception, 66 countries have joined the SUN movement (18). While the SUN movement primarily focuses on LMICs, challenges like non-communicable diseases, poor diet quality, and the impact of diet on climate change are also critical in high-income countries, highlighting the global nature of nutrition challenges.

#### 2.1.2 Mainstreaming nutrition

The Mainstreaming Nutrition Initiative (19) and the 2013 Nutrition for Growth Summit in London (20), shifted the focus from calorie adequacy to diverse, nutritious diets. At the summit, over 100 stakeholders pledged more than $4 billion for nutrition-specific projects and $19 billion for nutrition-sensitive initiatives (20). Nutrition’s integration into broader development agendas was further supported by the UN Decade of Action on Nutrition (2016–2025), led by the World Health Organization and the Food and Agriculture Organization, and supported by the World Food Programme, the International Fund for Agricultural Development, and the United Nations Children’s Fund (21).

During this period, organizations such as EAT (22) and the Consultative Group on International Agricultural Research (CGIAR) (23), emphasized nutrition-sensitive agriculture and sustainability. The first Global Nutrition Report in 2014 acted as an accountability mechanism, tracking progress against global nutrition targets (24). Notable initiatives during this period further included CGIAR’s work on improving access to affordable, nutritious, and diverse diets and the EAT Stockholm Food Forum, which fostered collaboration between business, science, and politics to achieve sustainable food systems.

#### 2.1.3 Sustainable development goals

The transition from the Millennium Development Goals (25) to the Sustainable Development Goals (SDGs) (26) in 2015 marked another pivotal shift, with a sharper focus on ending hunger and ensuring food and nutrition security. The SDGs called for a new food system paradigm, recognizing the importance to address all forms of malnutrition including underweight, micronutrient deficiencies as well as obesity, and underscoring the need for systems that support diverse, healthy diets. The Global Nutrition Report 2015 highlighted the intricate relationship between climate change and nutrition, and the pivotal role of businesses in addressing these challenges (27). By this time, 72 LMICs had reached the Millennium Development Goals target of halving the number of people suffering from hunger (28).

Based on one of the key recommendations from the nutrition community back in 2015 (29) the importance of measuring diet quality to connect agriculture and nutrition has been emphasized. As of October 2024, following provisional approval by the Inter-agency and Expert Group on SDG Indicators, indicators of diet quality (Minimum Dietary Diversity for non-pregnant women aged 15–49 years and for children aged 6–23 months) have been considered to be added to the SDG framework for the first time (30). This represents significant progress in the ability to measure and improve diet quality on a global scale, though they have yet to be fully approved (31).

#### 2.1.4 Multiplying impact

By 2017, the global community acknowledged that hunger was once again on the rise. The Global Nutrition Summit in Milan emphasized the powerful multiplier effect of improving nutrition across all SDGs, indicating that addressing nutrition is crucial to achieving broader development goals (32). The High-Level Panel of Experts report introduced a conceptual framework for food systems for diets and nutrition, reinforcing the need for integrated approaches (33).

#### 2.1.5 Integrating climate and diets

The 2019 United Nations Climate Action Summit and the United Nations High-Level Meeting on Universal Healthcare underscored the importance of integrating nutrition with climate action. The EAT-Lancet Commission contributed influential research on sustainable food systems, advocating for healthy diets that promote plant-based foods and reduce ultra-processed food consumption—bridging the gap between nutrition and environmental sustainability (34).

#### 2.1.6 Emphasizing data, accountability, and food systems transformation

From 2020 to 2024, there has been a strong focus on detailed data and accountability to improve global nutrition and food systems. The COVID-19 pandemic exposed the lack of resilience in food systems and highlighted diet quality as a key factor in COVID-19 co-morbidity, especially for individuals with non-communicable diseases. This underscored the need for robust food systems capable of withstanding crises and addressing both undernutrition and overnutrition.

The 2020 Global Nutrition Report underscored the importance of granular data to address nutrition inequalities effectively (35). Similarly, the Ceres2030 Report highlighted the need to refocus research on the needs of smallholder farmers, stressing the value of original data in shaping impactful interventions (36). In 2021, the United Nations Food Systems Summit, stressed multi-stakeholder approaches, policy coherence, and capacity building for systemic change (37). By 2024, these efforts culminated in the unveiling of a new economic model by the Food System Economics Commission. This model maps the impacts of two potential futures for the global food system, offering critical insights into the economic implications of different food system trajectories and guiding policy and investment decisions for a sustainable future (38).

#### 2.1.7 GAIN’s contributions to food systems transformation

In recent years, GAIN has played a key role in advancing global food system transformation through various initiatives. GAIN, in partnership with others, launched the Food Systems Dashboard, a tool that compares food system drivers, components, and outcomes across countries. The dashboard integrates data to identify challenges and strategies to improve nutrition, health, and environmental outcomes. As of 2024, it includes sub-national information for six countries, with two more forthcoming (39).

Building on the UN Food Systems Summit, GAIN introduced the Nourishing Food Pathways program, supporting food systems transformation in 10 countries to accelerate progress toward the Sustainable Development Goals, particularly SDG2 (40). GAIN also co-led the Food Systems Countdown Initiative, which launched baseline results to monitor food system transformation with data-driven insights (41). By June 2024, national data for 50 selected indicators became available through the dashboard to track food system progress. Through these initiatives, GAIN continues to drive progress toward more sustainable, equitable, and nutritious food systems globally, and provide exemplars for others to adopt or adapt.

### 2.2 A vision and opportunities for the future

For the future, achieving sustainable food systems will require an integrated approach that includes health, social protection, and water/sanitation systems. Addressing gender equity and recognizing the link between environmental sustainability and human health, particularly regarding climate and water, will be crucial for nutrition.

#### 2.2.1 Global initiatives—integrating nutrition and climate

Global climate and nutrition strategies remain misaligned. The Initiative on Climate Action and Nutrition (I-CAN) found that only 1% of climate-related Official Development Assistance financing and 2% of Nationally Determined Contributions mention nutrition explicitly or have concrete plans to address nutrition. Additionally, 95% of Global Nutrition Report commitments and 83% of public food procurement nutrition-related policies do not consider climate or sustainability (42).

Donors, United Nations agencies (such as the World Food Programme, International Fund for Agricultural Development, Food and Agriculture Organization, Children’s Fund, International Bank for Reconstruction and Development), and governments must integrate nutrition into their climate action plans and vice versa. I-CAN was launched at COP27 by the Egyptian presidency and is now implemented by GAIN alongside key UN partners, with the objective to bridge climate and nutrition strategies by providing baseline data, policy recommendations, and funding guidance, thus supporting countries in aligning their climate and nutrition goals (42). Other initiatives like Ceres2030’s food system roadmap (36), the Food Systems Countdown Initiative (43), and the Food System Economics Commission (44) should be locally adapted, using disaggregated data to ensure progress on both fronts.

In addition to its leadership of I-CAN, GAIN is actively supporting the implementation and expansion of Ceres2030 under Hesat2030 (45), providing greater focus on nutrition and climate impacts when developing country roadmaps for effective public and private sector interventions, as well as the Food Systems Countdown Initiative, to monitor food system progress. GAIN’s country dashboards further support this by offering sub-national evidence to inform tailored, effective food system transformations.

#### 2.2.2 National policies and food systems thinking

National policies remain fragmented, failing to integrate agriculture, nutrition, health, and climate. This policy incoherence can lead to contradictions, and policy goals in one area can undermine progress in another, for instance, when efforts to promote food systems transformation neglect the impacts of climate change (46). Despite commitments to food systems transformation, evidence on countries’ financing streams for these processes and active identification of policy incoherence remain limited (47).

Additionally, subsidies often do not support nutritious, climate-smart production, while rising food prices especially impact low-income households. While combining taxes on unhealthy foods with subsidies on healthy ones could be an effective approach (48, 49), there is a need to ensure that tax burdens do not disproportionately fall on low-income households (50). Additionally, shifting diets and supply chains to increase access to nutritious, low-environmental-impact foods is critical. Current research is focused on identifying and promoting culturally accepted, nutritionally valuable, and environmentally sustainable foods, with policy implications for dietary guidelines, agricultural incentives, public procurement, and social protection programs. It is also essential to consider the affordability of these foods, how their nutritional value may be influenced by women’s or children’s unique dietary needs, and how food consumption patterns vary across different regions and seasons.

GAIN supports national governments in defining their Food System Pathways and is actively developing a tool, recently piloted in Nigeria and now being deployed in nine additional countries, to help governments diagnose and improve the alignment of policies across different sectors (51). The Financial Flows to Food Systems tool, developed by the International Fund for Agricultural Development and the World Bank, provides data on food systems financing to inform policy decisions and promote transparency. GAIN is supporting its rollout in 11 countries (47). GAIN also fosters collaboration with the private sector through initiatives like the Nutrition Enterprise Development, which helps local entrepreneurs scale nutrition-focused businesses (52), and the Nutritious Foods Financing Facility, which provides financing to SMEs producing affordable, nutritious foods (53). Additionally, GAIN co-convenes the SUN Business Network, fostering private-sector commitments to reducing malnutrition through healthy food availability.

#### 2.2.3 Trade, dietary and production diversification

Trade policies will play a crucial role in addressing food and nutrition security, economic growth, and sustainability goals like climate change. There is a need for a nuanced understanding of the complex interactions (54) between these areas and a stronger focus on integrating nutrition and environmental considerations into trade policies (55).

Research should explore how climate change will affect nutritious food production and trade flows, with a focus on developing healthier crops beyond staples like wheat, maize, and rice. Emphasis on fruits, vegetables, and sustainable protein sources is key for diversifying diets and improving nutrition, especially in LMICs. Understanding the behavioral and cultural challenges of dietary changes is also vital, as education and culturally appropriate strategies are needed to promote healthier, more sustainable diets (56, 57).

GAIN is driving change at the consumer level through initiatives like Consumer Demand Generation, which encourages the purchasing of healthier, sustainable foods through social marketing and retail promotion strategies. The Food Culture Alliance aims to address food preferences, a determinant of consumer demand, by leveraging food cultures strategies, ensuring that food system changes are both culturally relevant and widely acceptable (58).

#### 2.2.4 Biodiversity

An analysis under the Initiative on Climate Action and Nutrition found that nutrition was generally not strongly integrated into 192 national biodiversity strategies (59). Biodiversity is essential for sustainable food systems, but diets lacking in diversity—especially plant biodiversity—continue to undermine both environmental sustainability and health outcomes. Recent research has highlighted the importance of adopting diets rich in plant biodiversity, such as the Mediterranean diet, which not only supports environmental sustainability but also enhances health (60).

Clear opportunities exist to find co-benefits through improved diversity in production and consumption, benefits to soil health (and, in turn, the nutritional content of crops), and food systems resilience, which helps to ensure sustained access to healthy diets even in the face of environmental and socio-economic challenges (59). Policy recommendations emphasizing the need to promote the integration of underutilized crops, support sustainable agricultural practices, and foster consumer awareness through education and clear food labeling are needed. This involves promoting the cultivation of crops that are well-suited to local conditions and more resilient to environmental stressors.

GAIN, as an active partner alongside the African Union, is involved in initiatives like the Vision for Adapted Crops and Soils, led by the U.S. Department of State. These initiatives aim to identify and promote public and private investments into the most nutritious and climate-resilient crops in Africa (61). Such strategies can help increase dietary diversity, improve nutritional outcomes, and contribute to the conservation of biodiversity, ultimately supporting more resilient and sustainable food systems globally.

#### 2.2.5 Technology innovations

Technology and innovation will drive advancements in nutrient-enriched crops and biofortification, which are essential for improving food quality, especially for vulnerable populations, and increasing crop resilience to environmental stressors. However, scaling these innovations remains a challenge.

Breeding programs need to focus on healthy diets and resilient crops, ensuring new varieties reach both farmers and consumers to support sustainable, climate-smart agriculture. The development of alternative animal source foods is another key area, with potential benefits for both health and the environment (62). However, deeper understanding is needed regarding their nutrition, processing, environmental impact, affordability, and food safety. Many types of alternative proteins require significant processing, which can contribute to environmental pressures. Striking a balance is essential to ensure these innovations support sustainability without unintended negative impacts.

GAIN, in collaboration with the Food and Agriculture Organization and other partners, is conducting research to ensure alternatives to animal source foods positively contribute to global food systems (including through nutrition, environmental sustainability, food safety, and socioeconomic considerations). Addressing these challenges will be pivotal in shaping sustainable, resilient, and equitable food systems over the coming years.

#### 2.2.6 Monitoring global diet quality

There has been a historical lack of mechanisms for monitoring diet quality globally, which is critical for developing effective nutrition policies and ensuring accountability in improving diets.

The future should focus on improving global diet quality monitoring and addressing diet disparities across different demographic groups. This will involve proposing new SDG diet quality indicators, such as Minimum Dietary Diversity for Women and Children, and using data to inform global and national policy interventions.

The Global Diet Quality Project (63), a collaboration between Gallup, Harvard University, and GAIN, has developed the first-ever mechanism for monitoring diet quality globally. Data collected from 85 countries representing 85% or the world’s population allow for an in-depth understanding of how diet quality varies across different demographics, providing crucial insights for developing effective nutrition policies and proposing new SDG diet quality indicators, such as Minimum Dietary Diversity for Women and Children.

## 3 Discussion

Reflecting on past efforts, certain missteps must be avoided as we move forward.

Actionable Indicators: While focusing on long-term outcomes, like stunting has raised awareness of nutrition and accelerated investments, it has often overshadowed more actionable, short-term, indicators like diet quality, which are easier to modify and better suited for tracking progress in the medium term.

Crop Breeding: Another critical area of past oversight was in crop breeding programs, which prioritized high yields over nutrient density and sustainability. This focus on quantity over quality contributed to the persistence of micronutrient deficiencies, especially in vulnerable populations. Future breeding efforts must emphasize nutrient-rich (or nutrient enriched, i.e., biofortified) crops that not only sustain high yields but also support healthy diets. The past emphasis on high yields led to intensive monoculture production systems that created significant environmental degradation, including loss of biodiversity, soil depletion, and unsustainable natural resource use. Future crop breeding must balance the need for greater productivity, with the need for higher nutrient density and environmental sustainability.

Food Systems Transformation: Despite progress, challenges persist in transforming food systems. Regions like Sub-Saharan Africa and South Asia still face significant hurdles in achieving nutritional security and climate resilience. These challenges are often compounded by limited funding, inadequate access to technology, and political instability, which hamper the implementation of effective interventions. Additionally, addressing micronutrient deficiencies and integrating nutrient-dense foods into everyday diets, remain under-addressed. The persistence of these issues can be attributed to a combination of factors, including insufficient financial resources, technological limitations, and a lack of supportive policy frameworks. Equity is vital to be considered and discussed, as new dietary recommendations and food system transformations are proposed, existing inequalities must be exacerbated. For instance, while alternative animal source foods may offer environmental and health benefits, they could also have unintended consequences for low-income populations, such as reducing access to affordable protein sources, increasing reliance on highly processed foods that contribute to overweight and obesity, or disrupting traditional livelihoods. Addressing these concerns requires a careful balance between promoting sustainable practices and ensuring that vulnerable populations are not disproportionately affected.

To achieve a truly sustainable transformation of global food systems, several key elements must be addressed:

1. Increased and sustained financial investment is essential to support research, development, and the scaling of innovative practices. Financial flows to food systems transformation need to be analyzed, for example, using the Financial Flows to Food Systems tool, to foster mutual accountability across both public and private sectors. Incentivizing private sector investment in sustainable practices through tax benefits, grants, or matched funding can further align efforts with sustainability and equity goals.
2. Coherent and integrated policy frameworks that align nutrition, climate, and equity goals are crucial. Such policies should provide clear guidance and incentives for adopting sustainable practices and support the transition to more resilient food systems. Tools such as the policy coherency tool, can help ensure that policies on agriculture, health, and environment reinforce each other.
3. Technological advancements, particularly in areas such as data collection, crop breeding, and sustainable farming techniques, are necessary to meet the challenges posed by climate change and evolving dietary needs. These innovations must be accessible and adaptable to different regional contexts. Scaling technology adoption could be supported through regional innovation hubs and training programs. Collaborations with tech firms and regional stakeholders could further make these technologies more affordable and regionally relevant.
4. The availability and accessibility of comprehensive, disaggregated data is vital for monitoring progress, identifying gaps, and making evidence-based decisions. Platforms like the Food System Dashboard as well as national efforts on sub-national data and monitoring initiatives can provide the insights needed to track and guide food system transformations effectively.
5. The transformation of food systems requires collaboration across a broad spectrum of actors, including governments, international organizations, the private sector, civil society, and local communities. Dedicated platforms and alliances can encourage knowledge sharing, align efforts toward common goals, and foster transparency. Establishing task forces focused on specific challenges can further help balance competing priorities and ensure a coordinated approach to food systems transformation.

## Data Availability

The original contributions presented in the study are included in the article/supplementary material, further inquiries can be directed to the corresponding author.
